# Unusual presentation of ‘internal hernia’ after robot-assisted radical cystectomy

**DOI:** 10.1093/jscr/rjae178

**Published:** 2024-03-21

**Authors:** Lucio Dell'Atti

**Affiliations:** Division of Urology, University-Hospital of Marche, Ancona, Italy

**Keywords:** hernia, cistectomy, robotic surgery, urinary diversion, complications

## Abstract

Here is presented the first case of internal hernia developing from the space between ureter and muscle fascia after robot-assisted radical cystectomy with uretero-cutaneostomy diversion. An 82-year-old man underwent robot-assisted radical cystectomy with uretero-cutaneostomy diversion for high-grade urothelial carcinoma (pT2). On the Postoperative Day 7, the patient presented abdominal pain and nausea. Abdominal computed tomography showed that a part of the small intestine was protruding between the right ureter and the transverse fascia, and was strangulated, causing an obstruction of the intestine. Patient underwent an emergency laparotomy that revealed prolapse and strangulation of the small intestine through the space between the right ureter and the transversalis fascia. The ischemic intestinal tract and ureter were resected. A new right uretero-cutaneostomy diversion anastomosis with use of ureteral stent single J was created. The man was discharged 28 days after surgery, and his clinical course was uneventful through follow-up.

## Introduction

Standard curative cystectomy is the gold standard in the treatment of bladder cancer. The type of urinary diversion (UD) depends on the patient’s general health condition, the specific health conditions, and the patient’s desire for a better life. In the USA and Europe, ileal conduit is still the most popular transplant method after radical cystectomy (RC) [1]. However, stoma problems remain a major problem and affect the quality of life after RC. These complications include skin erosion, stenosis, parastomal hernia, and stoma failure [2, 3]. Parastomal hernia is a common complication after robot-assisted radical cystectomy (RARC), occurring in 20% of patients and requiring surgical treatment in 15% of patients [2, 4]. The aim of this paper is to accentuate the importance of expert surgeon’s assessment on technical factors because outcomes depend on technique’s expertise. Here is the report on the first case of small bowel obstruction and internal herniation between right ureter and trasversalis fascia after RARC with UD.

## Case report

**Figure 1:**
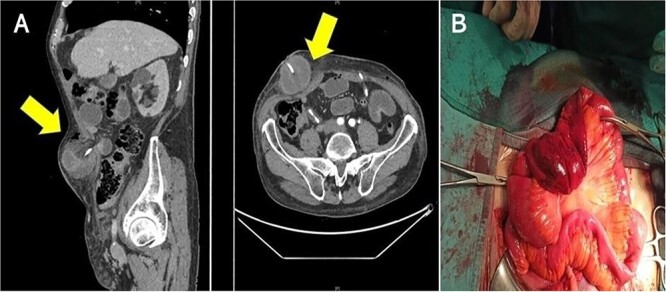
(A) Computed tomography shows the small intestinal herniation from the space between right ureter and trasversalis fascia; the arrows show hernial orifice; (B) surgeons observed engorgement of the bowel loops.

An 82-year-old white male (height: 174 cm, weight: 68 kg, body mass index: 25.3 kg/m^2^) presented with a history of gross hematuria that had been ongoing for 2 months. Cystoscopy revealed a large mass (3 × 2 cm) on the right wall of the bladder; computed tomography and MRI showed cT2bN1M0 bladder cancer. Medical records revealed that the patient received radical external beam radiation therapy for locally advanced prostate cancer 5 years ago and that he had abdominal surgery as an infant for appendectomy. His bladder tumor was removed with endoscopic technique and he was diagnosed a high-grade (pT2) urothelial carcinoma. He then underwent RARC with ureterocutaneous urinary diversion and pelvic lymph node dissection. On the seventh postoperative day, the patient complained of abdominal pain and nausea. Body temperature was 38.5°C. Physical examination revealed a distended abdomen and a large, irreducible, painful abdominal lump next to the right stoma diversion. Laboratory tests showed leukocytosis (21 000/ml) and increased C-reactive protein (19.58 mg/dl). Abdominal computed tomography showed that a part of the small intestine was protruding between the right ureter and the transverse fascia and was strangulated, causing an obstruction in the intestine (Fig. 1A). Emergency laparotomy revealed prolapse and strangulation of the small intestine through the space between the right ureter and the transversalis fascia, resulting in discoloration of the small intestine and ureter of interest (Fig. 1B). The strangulation was released, but there was no improvement in blood flow in sections of the small bowel strangulated, so intestinal resection and reconstruction was performed. The ischemic tract of the ureter was resected and a new right uretero-cutaneous anastomosis with use of ureteral stent single J (Ch 6) was created. The clinical course of the patient, who was discharged from the hospital 28 days after the surgery, remained stable during follow-up.

## Discussion

With advancing surgical technology, minimally invasive procedures have become the preferred mode of surgery over exploratory laparotomy for many urologic procedures. Advantages of minimally invasive surgery include decreased recovery time, length of hospital stay, and postoperative infection rate [1].

Guidelines recommend RC combined with pelvic lymphadenectomy for the treatment of aggressive bladder cancer. The incidence of complications after cystectomy can be very high. Most patients develop one or more complications; After RC, 20% require intervention and 20%–30% are admitted after discharge [2]. Complications are expensive, delay recovery, and increase mortality. Morbidity has been reduced due to centralization of services [3], advances in anesthesia and surgery, and faster recovery [4]. Given the prevalence of comorbid conditions, patients undergoing RC may benefit more from robotic surgery than others. Parekh *et al*. [3] reported that RARC was not inferior to open surgery in terms of cancer at 2 years. Postoperative hernia is a major complication of abdominal surgery. Estimates of the incidence of incisional ventral hernias vary depending on the size and location of incision; 10%–15% of patients undergoing open abdominal surgery will develop an incisional hernia, with midline location, vertical incision, and placement in the upper abdomen, all associated with higher risk of hernia [5]. Multiple studies have demonstrated that the incidence of trocar site hernia after minimally invasive surgery is lower than the incidence of incisional hernia after exploratory laparotomy, though estimates of this incidence vary widely. Studies of urologic minimally invasive procedures have valued the incidence of trocar site hernia at up to 1.3%. UCS was first described 40 years ago by Johnston as bilateral end-to-end cutaneous ureterostomy for the treatment of UD [6]. Transplantation of the ureter to the skin is the simplest method of UD and is the only transplant that does not require removal of a part of the digestive system. The main indications for UCS are planning for palliative care, presence of significant disease, poor quality of life, history of gastrointestinal RT, or the presence of pathologies that preclude the use of an intestinal segment (ulcerative colitis and Crohn’s disease) [7]. Complications, especially stoma necrosis, and stenosis are rare, and most patients require ureteral catheterization. The combination of RARC with UCS reduced the need for transfusion, length of hospital stay, and minor complications [5]. Small bowel obstruction resulting from internal herniation between ureter and muscle fascia is extremely rare after RC [6, 7]. There is no established protocol to prevent hernia after RARC combined with UD. It is thought that incisional hernias arise from the patient and his characteristics [8]. Patient characteristics include obesity, previous abdominal surgery, age, operations that increase bowel movement, such as postoperative constipation or cough, and other factors that affect wound healing, such as infection, diabetes, chemotherapy, and malnutrition [8, 9]. Technical factors are port position, trocar form, movements of robot arms, fascial closure technique, and operative time.

RARC it is a safe and effective procedure with a high success rate. However, as with any surgery, there are risks and potential complications that can arise. The case presented here highlights the importance of precise technique during robotic surgery and some of the aforementioned risks for technical factors. Correct placement of robotic trocars requires that the trocar be placed perpendicular to the abdominal wall so that the length of trocar within the abdominal wall is minimized. However, further research is needed to define appropriate stoma location, dimensions of ureteral anatomy, and guidelines for fascial closure.
